# Comprehensive Analysis of the GRAS Gene Family in *Paulownia fortunei* and the Response of DELLA Proteins to Paulownia Witches’ Broom

**DOI:** 10.3390/ijms25042425

**Published:** 2024-02-19

**Authors:** Yixiao Li, Yabing Cao, Yujie Fan, Guoqiang Fan

**Affiliations:** 1 College of Forestry, Henan Agricultural University, Zhengzhou 450002, China; l18338902058@163.com (Y.L.); cyb201406@163.com (Y.C.); fanyujie@henau.edu.cn (Y.F.); 2 Institute of Paulownia, Henan Agricultural University, Zhengzhou 450002, China

**Keywords:** *Paulownia fortunei*, PaWB, GRAS, DELLE, dwarf, GA3

## Abstract

The *GRAS* (GAI\RGA\SCL) gene family encodes plant-specific transcription factors that play crucial roles in plant growth and development, stress tolerance, and hormone network regulation. Plant dwarfing symptom is mainly regulated by DELLA proteins of the *GRAS* gene subfamily. In this study, the association between the *GRAS* gene family and Paulownia witches’ broom (PaWB) was investigated. A total of 79 *PfGRAS* genes were identified using bioinformatics methods and categorized into 11 groups based on amino acid sequences. Tandem duplication and fragment duplication were found to be the main modes of amplification of the *PfGRAS* gene family. Gene structure analysis showed that more than 72.1% of the *PfGRASs* had no introns. The genes *PfGRAS12/18/58* also contained unique DELLA structural domains; only *PfGRAS12*, which showed significant response to PaWB phytoplasma infection in stems, showed significant tissue specificity and responded to gibberellin (GA3) in PaWB-infected plants. We found that the internodes were significantly elongated under 100 µmol·L^−1^ GA3 treatment for 30 days. The subcellular localization analysis indicated that *PfGRAS12* is located in the nucleus and cell membrane. Yeast two-hybrid (Y2H) and bimolecular fluorescence complementation (BiFC) assays confirmed that PfGRAS12 interacted with PfJAZ3 in the nucleus. Our results will lay a foundation for further research on the functions of the *PfGRAS* gene family and for genetic improvement and breeding of PaWB-resistant trees.

## 1. Introduction

Plants have evolved a complete and complex set of regulatory mechanisms during long-term adaptation to their environments. The *GRAS* (GAI\RGA\SCL) gene family encodes transcription factors that are found in most higher plants where they play crucial roles in many biological processes, including developmental processes, light signal transduction, gibberellin (GA) signal transduction, stem meristem maintenance, and axillary meristem formation in plants [1]. All the GRAS proteins, excluding the DELLA subfamily, exhibit high variability at the N-terminus. The N-terminal structural domains of the *GRAS* gene family are found within short easily interacting fragments in intrinsically disordered regions (IDRs) [2]. These domains fold specifically upon encountering suitable ligands and are significant contributors to gene-specific functions [1]; nowadays, it has been studied and reported in several species such as *Dendrobium catenatum* [3], *Gossypium hirsutum* L. [4], and *Oryza sativa* L. [5].

DELLA proteins are negative regulators of the GA pathway that are largely responsible for plant dwarfing [6]. The unique motifs of DELLA proteins allow them to interact with specific transcription factors in hormone regulatory pathways, such as the jasmonic acid (JA) [7], salicylic acid (SA) [8], and brassinosteroid (BR) [9] signaling pathways. Such interactions form regulatory networks that regulate plant defense responses and cause a range of symptomatic changes in plants [10]. DELLA proteins can competitively bind with jasmonate-zin-domain protein (JAZ) and MYC2 transcription factors, leading to the release of MYC2 and activation of the JA defense pathway [1]. GA triggers the degradation of DELLA proteins, which promotes plant growth [11]. Mutations in the DELLA structural domain can cause a semi-dominant GA-insensitive dwarf phenotype [12,13]. Paulownia witches’ broom (PaWB) is accompanied by severe symptoms of internode shortening. However, the relationship between DELLA proteins and the dwarfing symptom of PaWB is still unknown.

*Paulownia fortunei* (*P. fortunei*) is widely cultivated in China because of its high adaptability and rapid growth [14], which is of great significance to China’s agroforestry economic production and ecological environment regulation. However, the infestation of PaWB phytoplasma causes a variety of diseases such as dwarfing and clumping of branches, which in severe cases can directly cause the death of the trees, which seriously restricts the industrial development of *P. fortunei*. Phytoplasma is a group of Gram-positive bacteria belonging to Candidatus Phytoplasma, which can only survive on living cells due to its lack of a large number of biochemical and metabolic pathways, and does not infect animals, but can cause disease in more than 1000 plants worldwide [15]. PaWB phytoplasma specifically parasitizes *P. fortunei*, which affects the growth of branches and trunks and the shape of the trunk, leading to the loss of economic wood. Because in vitro cultivation of PaWB phytoplasma is difficult, the real cause of PaWB is still unclear. With the development of third-generation sequencing technology, our group’s successful assembly of the *P. fortunei* genome and the PaWB phytoplasma genome provides essential information for the study of PaWB at the gene level, and it is urgent to identify PaWB-related genes for the breeding of PaWB-resistant plants. We are talking about a family of GRAS genes that has been reported to be widely involved in plant growth and development, stress response, and hormone signaling [16]. Especially, DELLA proteins are an important factor affecting plant height [6]. Currently, whether effectors affect DELLA proteins and thus regulate dwarfing symptoms has not been investigated in *P. fortunei*. Therefore, in this study, we characterized for the first time the relationship between DELLA proteins and PaWB. These findings will contribute to an understanding of the relationship between the *P. fortunei* defense mechanisms and the GRAS proteins and provide a basis for genetic breeding in forestry.

## 2. Results

### 2.1. Identification Analysis of the PfGRAS Gene Family

**Figure 1:**
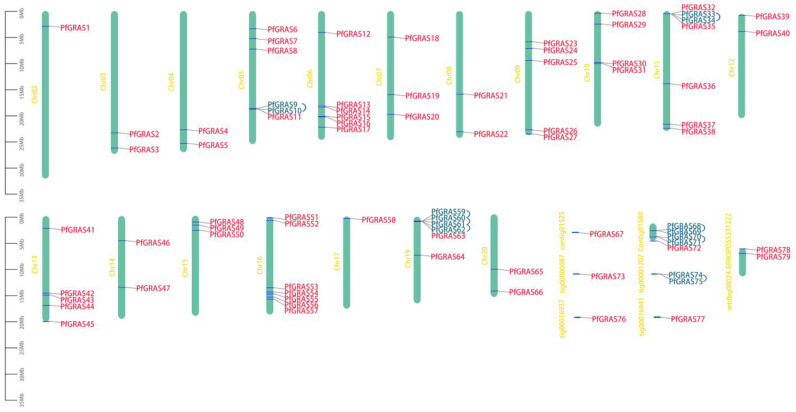
Chromosome distribution of *PfGRAS* gene family members. Vertical bars indicate the chromosomes. Tandem duplication genes are highlighted in blue and connected by blue lines. The left scale represents chromosome length.

After screening, a total of 79 *PfGRAS* genes were identified and named from *PfGRAS1* to *PfGRAS79* in the order they were arranged on the chromosomes (Figure 1). The predicted viscosity average molecular weight (MV), aliphatic index, and isoelectric point (pI) of the encoded proteins were 23.45–87.15 kDa, 66.25–108.3, and 4.64–9.18, respectively. The pI analysis showed that most of the *PfGRASs* were acidic proteins; the exception was *PfGRAS75*, which was a basic protein. Eight *PfGRASs* with instability indexes <40 were classified as stable proteins; the remaining proteins were classified as unstable, and all of them were hydrophilic. Subcellular localization prediction indicated that 62% of the *PfGRASs* were located in the nuclear region, 16% were in the cytoplasm, and only a few were in the chloroplast or endoplasmic reticulum. These findings suggest that the PfGRAS transcription factors regulate the expression of downstream target genes mainly in the nucleus (Table S1).

### 2.2. Phylogenetic and Collinearity Analysis of the PfGRAS Gene Family

**Figure 2:**
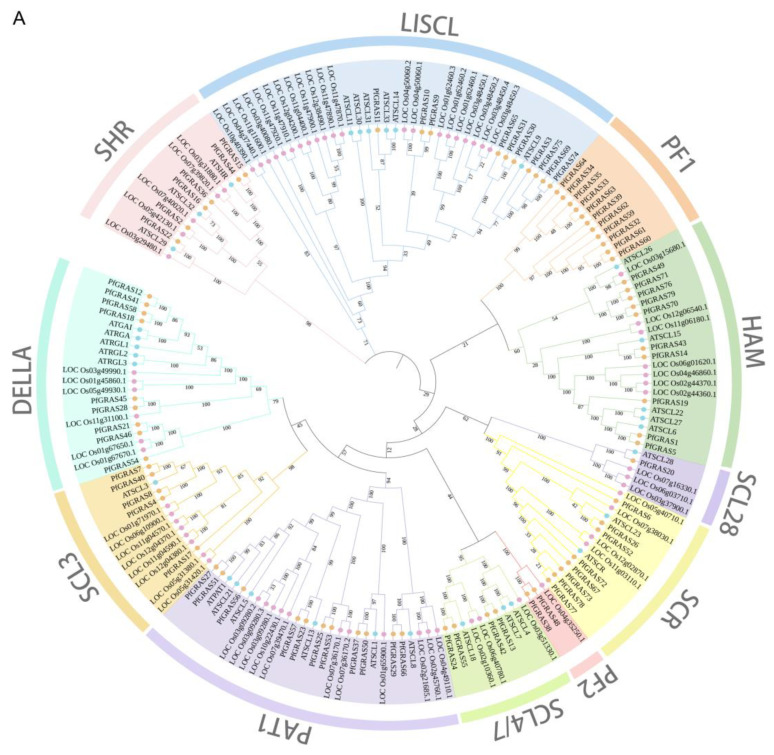

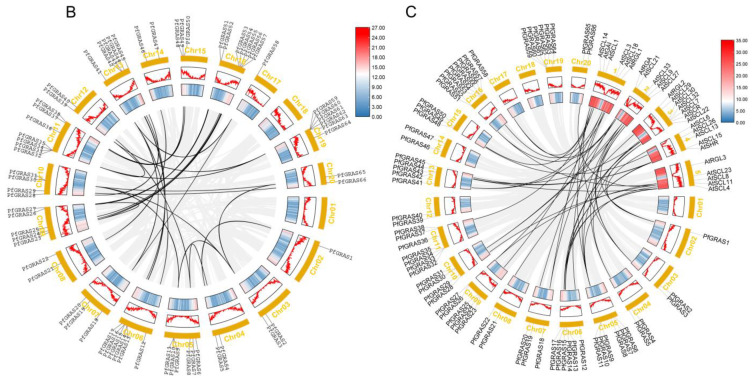
Phylogenetic and collinearity analysis of *PfGRAS* gene family members. (**A**) The phylogenetic tree of *pfGRAS*, *AtGRAS*, and OsG*RAS* divide the members into 11 groups. (**B**) The black lines represent the collinearity gene pair in the *Paulownia fortunei* (*P. fortunei*). The colored boxes represent gene density. (**C**) Collinearity analysis between *Arabidopsis thaliana* (*A. thaliana*) and *P. fortunei.* The black lines refer to the collinear gene pair in the *A. thaliana* and *P. fortunei*.

The *PfGRAS* gene’s amino acid sequences together with the GRAS amino acid sequences of the monocotyledon *Oryza sativa* L. (69 members) and the dicotyledon *Arabidopsis thaliana* (*A. thaliana*) (32 members) were used to construct a phylogenetic tree. Prior to phylogenetic reconstruction, *PfGRAS* genes that contained <300 amino acids were excluded to ensure the reliability of the analysis [17]. The *PfGRASs* formed 11 clusters in the phylogenetic tree, namely SHR, LISCL, HAM, SCL28, SCR, SCL4/7, PAT1, DELLA, SCL3, PF1, and PF2 (Figure 2A). None of the *PfGRASs* clustered with the DLT and LS subfamilies of *A. thaliana*, and the PF1 and PF2 subfamilies did not include any *AtGRASs*. The PF1 subfamily formed a distinct monophyletic branch, suggesting that this subfamily was either acquired in the *P. fortunei* lineage or disappeared completely from the *A. thaliana* and *Oryza sativa* L. lineages after they diverged from their latest common ancestor. We hypothesize that the PF1 subfamily may have a unique role in the adaptive evolution of *P. fortunei*.

To further explore the evolutionary relationships of the *PfGRAS* gene family, we identified 14 *PfGRAS* genes that were clustered in 5 tandem repeat event regions (Figure 1). These 14 genes all belonged to the LISCL and PF1 subfamilies, which were the 2 largest subfamilies in the phylogenetic tree, suggesting that the LISCL and PF1 subfamilies may have played important roles in the expansion of *PfGRASs*. There were 34 fragment repetitions among the 49 members involved simultaneously (Figure 2B). Several of the repetitions were one-to-many events, possibly due to gene expansion in the same subfamily. Because *A. thaliana* is a model plant that has been widely studied, most of the genes in *A. thaliana* have been functionally characterized. Therefore, we used the *A. thaliana* data to find homologous *P. fortunei* genes that may be important for studying the evolutionary history of *PfGRASs*. We found 44 incidences of fragment repetition events between 27 *AtGRAS* genes and 58 *PfGRAS* genes, with each *AtGRAS* gene having 1 to 3 homologous *PfGRAS* genes (Figure 2C). Therefore, we suggest that tandem repetition and fragment duplication events may have played a significant role in the evolutionary expansion of the *PfGRAS* gene family.

### 2.3. Conserved Motifs and Gene Structure of the PfGRAS Gene Family

**Figure 3:**
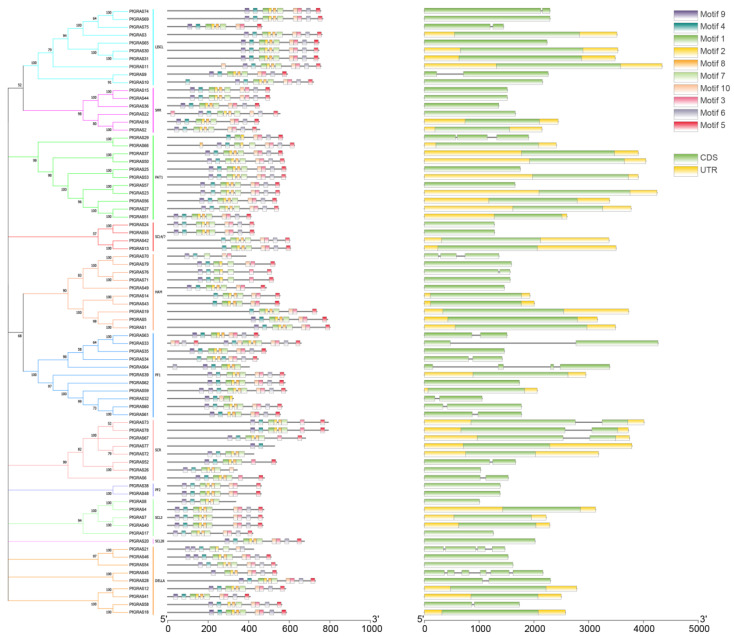
Phylogenetic groups, motif compositions, and gene structures of *PfGRAS* gene family. MEME analysis revealed a schematic representation of the conserved motifs among the *PfGRAS* gene family. Each color represents a distinct motif; gene structures of the *PfGRAS* gene family are depicted with the coding sequences (CDS) and untranslated regions (UTR) appearing in green and yellow boxes, respectively.

We identified conserved motifs in all the PfGRAS proteins using MEME and found that most *PfGRAS* genes in the same subgroup had similar motifs. For example, all 6 *PfGRAS* genes in the SHR subfamily contained a full set of 10 motifs in the same order in the sequences. We also found that the motifs tended to be located at the C-terminus, supporting the view that the C-terminal regions of GRAS proteins were more conserved than the N-terminal regions [18]. Gene structure analysis showed that 72.1% of the *PfGRAS* genes had no introns. Among the 22 *PfGRAS* genes that had introns, the number of introns ranged from 1 to 5, and some *PfGRAS* genes in the same group in the phylogenetic tree had similar exon-intron structures. Most homologous pairs also had conserved exon–intron structures. However, some *PfGRAS* genes in the same group exhibited significant diversity in the exon–intron structure. It is possible that some of these gene pairs experienced intron loss or acquisition events during the evolutionary process (Figure 3).

### 2.4. Sequence Alignment and Cis-Element Analysis of the PfGRAS Gene Family

**Figure 4:**
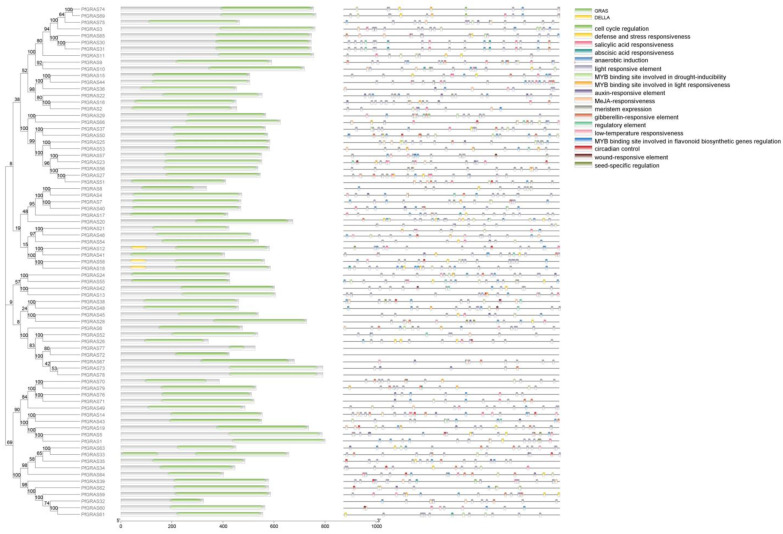
Structural domain and cis-acting element analysis of the *PfGRAS* gene family. Green and yellow colors represent the GRAS and DELLA structural domains, respectively; different colors represent different cis-acting elements.

The domains varied among the 79 PfGRAS proteins in two main ways. Some *PfGRASs* (e.g., *PfGRAS33*) contained multiple GRAS domains. In *Oryza sativa* L., *OsGRAS39* and *OsGRAS54* were annotated as having two or three domains [19], possibly because of tandem duplication events [20]. Other *PfGRAS* genes (e.g., *PfGRAS47*) contained only one GRAS domain in the full-length sequence. Members of the DELLA subfamily, *PfGRAS21/46/54/45/28*, clustered with DELLA proteins in a single branch in the phylogenetic tree. Domain analysis indicated that these PfGRASs lacked DELLA and TVHNVP domains; otherwise, they may have a close phylogenetic relationship with DELLA proteins. Additionally, 18 different cis-acting elements were predicted in the promoter regions of the 79 *PfGRAS* genes. The 79 promoters all contained light-responsive elements, indicating that light-response, stress, and hormone-response elements play important roles in the transcription of *GRAS* genes (Figure 4).

### 2.5. Diversified Expression Patterns of the PfGRAS Gene Family

**Figure 5:**
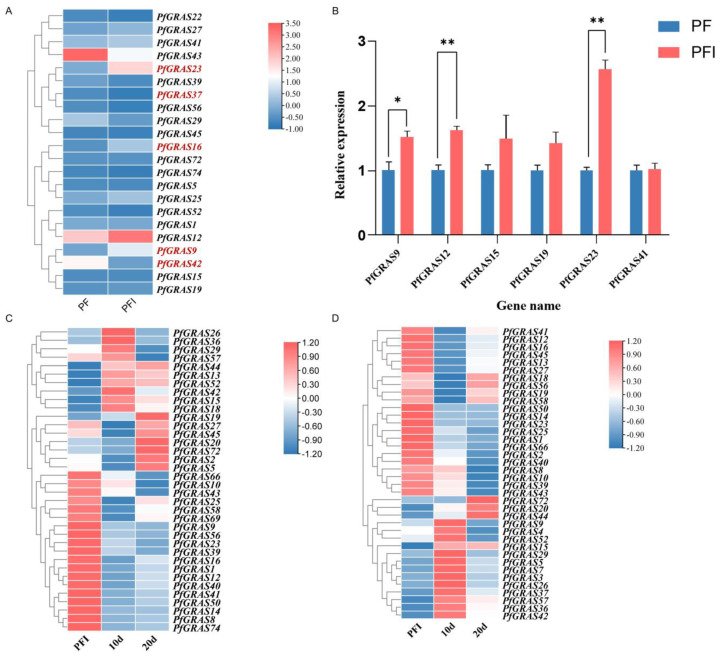
Expression analysis of *PfGRAS* gene family. (**A**) Heatmap of *PfGARS* gene expression in response to PaWB phytoplasma. The gene for |log2FC| > 1.5 is highlighted. (**B**) qRT-qPCR of *PfGRAS* gene expression in response to PaWB phytoplasma. Significant and highly significant differences compared with the gene expression in PF are shown as * (*p* < 0.05) and ** (*p* < 0.01), respectively. (**C**) Heatmap of *PfGRAS* genes expression in response to methyl methanesulfonate (MMS) in PFI; 10(20) d, PFI seedlings treated with MMS for 10(20) days. (**D**) Heatmap of *PfGRAS* gene expression in response to rifampicin (Rif) in PFI; 10(20) d, PFI seedlings treated with Rif for 10(20) days. In the heatmaps (**A**,**C**,**D**), scaled log2FC expression values based on transcriptomics data are shown from blue to red, indicating low to high expression.

To identify candidate PaWB-related *PfGRAS* genes, we analyzed the expression changes of the 79 *PfGRASs* using transcriptome data. We detected 52 *PfGRASs* that were differentially expressed between PF and PFI, and 17 of them were significantly differentially expressed (Figure 5A). *PfGRAS9/12/15/16/19/23/25/27/41* were significantly up-regulated (log2FC > 0.7), and *PfGRAS22/29/37/39/42/43/52/56* were significantly down-regulated in PFI (log2FC < −0.7). To further evaluate the relationship between *PfGRAS* genes and PaWB, we performed a statistical analysis of the expression changes of the *PfGRAS* genes in plants treated with high concentrations of methyl methanesulfonate (MMS) and rifampicin (Rif). Among them, *PfGRAS1/8/10/12/14/16/23/25/39/40/41/43/50/58/66* were co-up-regulated of MMS and Rif treatment, whereas *PfGRAS5/15/20/26/36/42/44/52/72* were co-down-regulated of MMS and Rif treatment (Figure 5C,D). These response trends imply that *PfGRAS12/16/23/41/50* may be the pathogenic genes related to the occurrence of PaWB, and *PfGRAS15* may be associated with PaWB inhibition. Finally, we randomly selected six differentially expressed *PfGRASs* for verification by qRT-PCR. The qRT-PCR results were consistent with the results obtained by transcriptome data analysis, indicating that the expression data obtained by transcriptome analysis were highly reliable (Figure 5B).

### 2.6. Specific Expression and Expression Analysis in Response to GA3 of the DELLA Subfamily

**Figure 6:**
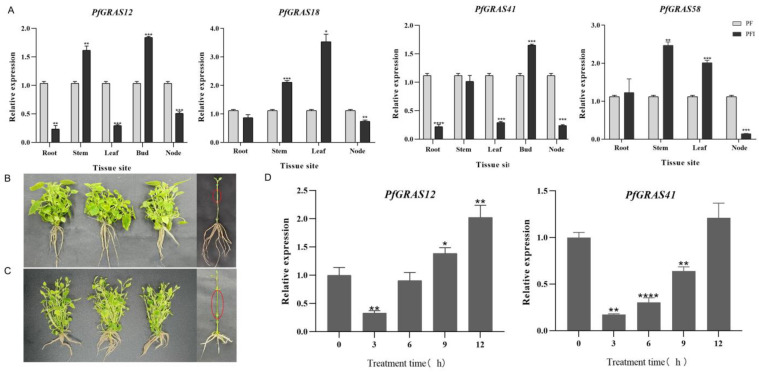
Tissue specificity and expression patterns of DELLA proteins under gibberellin (GA3) treatment. (**A**) RNA from roots, stems, leaves, buds, and nodes of PF and PFI grown for 30 days were extracted for tissue-specific validation, and *PfGRAS18/58* was not expressed in the buds. The white and black bars indicate the amount of expression in PF and PFI at different tissues; (**B**) PFI grown for 30 days as a control; (**C**) PFI treated with 100 µmol·L^−1^ GA3 for 30 days; (**D**) the gray bars indicate the amount of expression in the PFI at different treatment times. Significant and highly significant differences compared with the gene expression in PF are shown as * (*p* < 0.05), ** (*p* < 0.01), *** (*p* < 0.001), and **** (*p* < 0.0001), respectively.

It has been shown that DELLA proteins (RGA1) interact with PFI3, PIF4, and PIF8 of the PIF (phytochrome interacting factor) family to regulate GA-induced hypocotyl elongation in *A. thaliana* [21,22]. In this study, homology analysis revealed that four genes (*PfGRAS12/18/41/58*) were highly homologous to DELLA (RGA1) (Figure 2A). Under PaWB phytoplasma stress, the expression levels of *PfGRAS12*, *PfGRAS18*, and *PfGRAS58* were significantly up-regulated in stems, the expression levels of *PfGRAS12* and *PfGRAS41* were down-regulated and that of *PfGRAS58* was significantly up-regulated in roots and leaves, and the expression levels of four DELLA genes were down-regulated in nodes (Figure 6A). This demonstrates the tissue specificity of DELLA proteins in *P. fortunei*. We investigated the relationship between the shortening of internodes caused by PaWB phytoplasma infection and DELLA proteins by treating PFI with 100 µmol·L^−1^ GA3 for 30 days. We found that the internodes were significantly elongated (Figure 6B,C). Furthermore, the expression levels of *PfGRAS12/41* were significantly down-regulated after 3 h of treatment. Possibly due to the elimination of GA3, the expression gradually increased as the treatment time increased, reaching a peak at 12 h (Figure 6D). However, the expression of *PfGRAS18* failed to significantly respond to GA3 treatment, and *PfGRAS58* did not conform to the trend of DELLA protein response to GA3 (Figure S1). These results show that GAs can compensate for the dwarfing symptoms caused by PaWB phytoplasma, and *PfGRAS12/41* responded significantly to GA3 treatment under the PaWB phytoplasma infection.

### 2.7. Subcellular Localization of PfGRAS12

**Figure 7:**
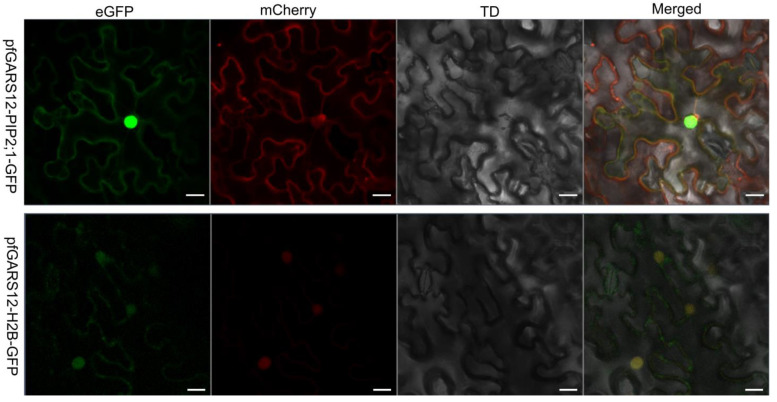
Subcellular localization of *PfGRAS12*; 35S: *PfGRAS12*-GFP construct was individually injected into the epidermal cells of *Nicotiana benthamiana* (*N. benthamiana*). The transient expression of *PfGRAS12*-GFP was observed and captured by a confocal laser scanning microscope. mCherry-PIP2;1 as a cell membrane marker. mCherry-H2B as a nucleus marker. Scale bars were 20 µm.

Because *PfGRAS18/58* did not significantly respond to GA3 treatment, PfGRAS41 had a C-terminal deletion of the typical DELLA structural domain and did not significantly respond to PaWB phytoplasma in stems, we selected *PfGRAS12* for further study. To substantiate the subcellular localization of the PfGRAS12 protein, we built a temporary expression construct. The *PfGRAS12*-GFP fluorescence signal was found on the cell membrane and in the nucleus (Figure 7). Therefore, we considered that the PfGRAS12 transcription factor gene had a downstream transcriptional regulatory role in the nucleus and cell membrane in response to PaWB phytoplasma infection. 

### 2.8. PfJAZ3 Is the Binding Partner of PfGRAS12

**Figure 8:**
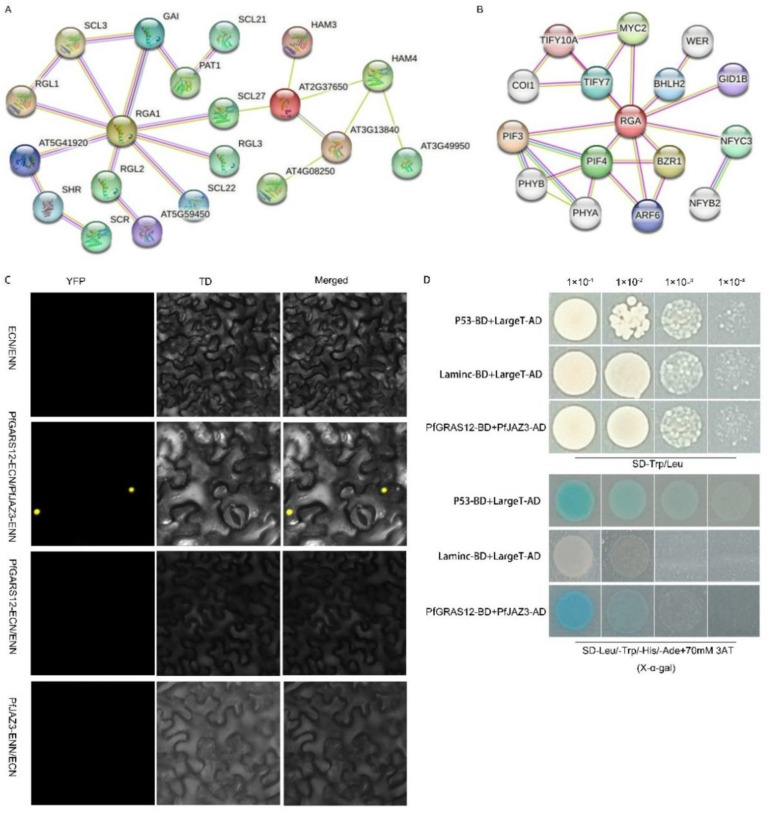
Prediction and validation of proteins interactions. (**A**) Prediction of *PfGRAS* gene family protein interactions; (**B**) prediction of interacting proteins with RGA1 in *A. thaliana*; (**C**) BiFC experiments verify the interaction between PfGRAS12 and PfJAZ3; (**D**) yeast two-hybrid verifies the interaction between PfGRAS12 and PfJAZ3.

Protein prediction indicated that 16 PfGRAS proteins may interact in *P. fortunei* (Figure 8A), and DELLA proteins interacted with JAZ3 (jasmonate-ZIM-domain protein 3) of the TIFY (TIF[F/Y] XG) family using STRING (Figure 8B). As a regulatory protein of the JA pathway [23], we considered whether JAZ3 might act together with DELLA proteins to regulate dwarfing symptoms of PaWB. To test this conjecture in *P. fortunei*, we identified the JAZ3 gene in *P. fortunei* that had the highest homology to *AtJAZ3* and named it *PfJAZ3*. In a previous study, it was shown that 70 mmol·L^−1^ 3-aminotriazole can completely inhibit the self-activation of the PFGRAS12 transcription factor [24]. A yeast two-hybrid assay indicated that PfGRAS12 interacted with PfJAZ3 in *P. fortunei* under 70 mmol·L^−1^ 3-aminotriazole inhibition of PfGRAS12 self-activation (Figure 8D). The assay also showed that JAZs were localized in the nucleus [23]. The BiFC assays confirmed the interaction of PfGRAS12 and PfJAZ3 in the nucleus (Figure 8C). These results suggest that DELLA-JAZ3, as a negative regulator of the GA-JA pathway, may be involved in the balance between plant growth and development and stress response under PaWB stress, which provides a new perspective for further research on the dwarfing symptoms caused by PaWB.

## 3. Discussion

GRASs play important roles in various developmental processes in plants, including seed germination, stem elongation, and leaf expansion [25,26,27]. In PFI, externally applied GA3 induced internode elongation, and *PfGRAS12* significantly responded to GA3. This finding, combined with the result that the GA4 and GA9 content was significantly lower in PFI than it was in PF [24], suggests that *PfGRAS12* is involved in the GA regulatory pathway under PaWB infestation. JA is a lipid-derived hormone molecule that responds to stressors, such as pathogens, herbivores, and abiotic stressors, while inhibiting plant root and hypocotyl growth [23]. Enhanced JA signaling was reported to promote the degradation of JAZ proteins, releasing DELLA proteins that negatively regulated the downstream PIF3 transcription factor and inhibited hypocotyl elongation [28]. DELLA proteins can also form a complex with PfPIF8 and PfSWP73 to co-regulate the expression of PfCYP714A, thereby promoting the development of dwarfing symptoms of PaWB [24]. DELLA proteins form an interplay linkage with JAZs that connects JAs and GAs at the transcriptional level, thereby influencing plant growth, development, and defense homeostasis [29]. In *P. fortunei*, DELLA and JAZ3 proteins interacted in the nucleus, jointly participating in the complex developmental and defense regulation of *P. fortunei*. This DELLA–JAZ3 interaction provides a new perspective on the regulatory mechanism of the dwarfing symptom of PaWB.

Because of the lack of variants or suitable transgenic platforms for forest research, the function and regulatory mechanisms of GRASs are still unclear. Studies into the functions of GRASs in woody plants have relied mainly on expression analysis and bioinformatics prediction. GRAS proteins exhibit functional specificity. In *Oryza sativa* L, the *AtLS* homologous gene *OsMOC1* was found to be involved in tillering regulation, leading to an increase in tillers and dwarfism in rice plants overexpressing *MOC1*. However, the *MOC1* deletion mutant lacked the main branch and did not lead to tillering [30]. *PfGRAS55/24* were significantly similar to *AtLAS*, suggesting that these genes may regulate the branching phenotype in PaWB. Conversely, *PAT1* and *SCL21* have been shown to promote hypocotyl elongation during yellowing [31]. We found that the expression levels of *PfGRAS25* and *PfGRAS27*, homologous genes to *PAT1* and *SCL21*, were significantly up-regulated in PFI, suggesting that *PfGRAS25/27* may control the basic developmental processes of *P. fortunei* under PaWB stress through light signals. SHR (SHORT ROOT) and SCR (Scarecrow) proteins act as positive regulators in radial pattern formation in roots and stems. SHR proteins were shown to regulate the elongation of hypocotyls in yellowed *A. thaliana* [32], and DELLA proteins have been shown to form an SCR–SHR–DELLA complex that is involved in the radial morphogenesis of roots and stems [33,34]. *PfGRAS15/44* are highly homologous to SHR, and *PfGRAS72/26* are highly homologous to SCR, suggesting that these genes may encode proteins that form complexes with DELLA proteins and participate in the formation of the dwarfing symptom of PaWB.

Changes in plant morphology resulting from alterations in gene expression levels may be the underlying cause of PaWB, with changes in plant hormone levels caused by the interaction of effectors, with plant proteins being the direct cause. Increases and decreases in hormone levels are the direct outcomes of plant metabolism. Following infection with PaWB phytoplasma, numerous substances in *P. fortunei* are affected and altered. The GRAS gene family is one of the key hubs in plant hormone regulatory networks, with the variable N-terminal domain of GRAS proteins providing a favorable prerequisite for hormonal linkage. As the primary negative regulator of the GA3 transduction pathway [35], DELLA proteins have been shown to play a crucial role in stem elongation. Therefore, the genome-wide identification and analysis of the GRAS gene family related to PaWB using bioinformatics methods, coupled with the analysis of changes in DELLA proteins during the occurrence of PaWB, provides a foundation for understanding the regulatory mechanisms underlying the dwarfing symptom of PaWB.

## 4. Materials and Methods

### 4.1. Plant Materials and Growth Conditions

All plant samples utilized in this study were from Henan Agricultural University’s Laboratory of Forestry Biotechnology in Zhengzhou, Henan Province, China. PF and PFI seedlings were selected and cultivated in 1/2 MS (Murashige and Skoog basal medium). The conditions for the culture were as follows: temperature 25 ± 2 °C, light intensity 130 μmol·m^−2^s^−1^, and light duration 16 h·d^−1^. After growing for 30 days, the roots, stems, leaves, buds, and nodes from PF and PFI were collected for further study.

For GA3 treatment, PFI with a root length of about 1 cm was transferred to 100 µmol·L^−1^ GA3 and normal medium (control). The stems were collected at 0, 3, 6, 9, and 12 h after transformation, flash-frozen in liquid nitrogen, and stored at −80 °C for RNA extraction. Three biological replicates were established for each treatment.

### 4.2. Identification and Chromosomal Location Analyses of PfGRAS Gene Family

The GRAS protein sequences of *A. thaliana* and *Oryza sativa* L. were used as query sequences to search the TAIR (TAIR-Home Page (arabidopsis.org)) and NCBI (National Center for Biotechnology Information (nih.gov)) databases to identify GRAS proteins of *P. fortunei*. Bidirectional blast alignment was performed using TBtools to identify homologous proteins of *A. thaliana* and *Oryza sativa* L. [36]. We downloaded the Hummer of GRAS (PF03514) from the plant transcription factor database (PlantTFDB) (PlantTFDB-Plant Transcription Factor Database@ CBI, PKU (gao-lab.org)). We searched the *P. fortunei* genome databases using hmmsearch with an E-value <0.05 to retrieve *P. fortunei* gene sequences. Finally, we used the NCBI-CDD (Conserved Domains Database (CDD) and Resources (nih.gov)) to identify conserved structural domains in the GRAS protein sequences, then removed sequences that did not contain the conserved structural domain. The remaining sequences were considered to be members of the PfGRAS gene family. The ProtParam tool (Expasy-ProtParam tool) was used for protein physicochemical analysis.

Based on the *P. fortunei* gene annotation data, the location of each *PfGRAS* gene in the genome was determined and named in the order they were arranged on the chromosomes.

### 4.3. Phylogenetic, Motifs, and Gene Structure Analysis of the PfGRAS Gene Family

To determine the phylogenetic relationships of PfGRAS proteins, we performed a multiple sequence alignment using ClustalW with default parameters. A total of 32 AtGRAS, 69 OsGRAS, and 77 *PfGRAS* genes were included in the alignment. A phylogenetic tree that included the 178 translated full-length GRAS amino acid sequences was constructed using the maximum likelihood method in MEGA 7.0 with 1000 ultrafast bootstrap repeats and analyzed using IQ-tree. The evolutionary tree was annotated and visualized using iTol (iTOL: Interactive Tree of Life (embl.de)).

We used the genome and annotation data of *P. fortunei* for gene structure analysis using TBtools. Conserved motifs were identified using MEME (MEME-Submission form (meme-suite.org)).

### 4.4. Cis-Acting Element and Collinearity Analysis of the PfGRAS Gene Family

TBtools was used to extract the promotor regions of the *PfGRAS* gene 2000 bp upstream of the transcription start sites. The promoter sequences were analyzed using PlantCARE (PlantCARE, a database of plant promoters and their cis-acting regulatory elements (ugent.be)) to predict possible cis-acting elements. *A. thaliana* data were downloaded from PlantTFDB based on the in-house genomic and gene annotation data of *P. fortunei*. One Step MCScanX was used to analyze covariance within *P. fortunei* and between *P. fortunei* and *A. thaliana*.

### 4.5. Gene Expression Analysis

Total RNA was extracted from the buds of 30-day-old *P. fortunei* (PF) and PaWB-infected *P. fortunei* (PFI) seedlings using a plant RNA extraction kit (Beijing Apbiotech Co., Ltd., Beijing, China). cDNA was synthesized using StarScript III All-in-one RT Mix with gDNA Remover (GenStar, Beijing), and diluted to 200 µg·mL^−1^. Primers were designed based on sequences from the NCBI database (S2). Expression levels were quantified using SYBR Green qPCR Premix reagent and qRT-PCR. The reaction system and amplification program were according to Cao et al. [37]. The *PfActin* gene was used as the internal reference, and each sample was replicated independently three times with both biological and technical duplicates. The 2^−ΔΔCt^ method was used for expression level analysis, and the statistical analyses were performed using GraphPad Prism8.

For tissue-specific analysis of DELLA subfamily members and their response to GA3, RNAs were extracted from the stems, roots, leaves, apexes, and nodes of 30-day-old PF and PFI seedlings. Additionally, RNAs were extracted from the stems of PFI seedlings treated with GA3 for 0, 3, 6, 9, and 12 h for qRT-PCR validation. The methods employed were the same as mentioned previously.

### 4.6. Vector Construction and Subcellular Localization

*PfGRAS12* was cloned using the KOD DNA polymerase and ligated into a PSAK277 expression vector that contained a green fluorescent protein (GFP) label, in which PF apical bud RNA was used as a template for amplification, and the pSAK277 vector was cleaved using FD-ECOR1 mono enzyme. The resulting recombinant plasmid, 35S: pSAK277-*PfGRAS12*-eGFP, was transformed into GV3101-Psoup-P19 for transient expression in *N. benthamiana* leaves. Subsequently, the transformed *N. benthamiana* plants were cultured at 25 °C for 72 h. Confocal laser scanning microscopy (Zeiss LSM 710, Göttingen, Germany) was used to visualize and capture eGFP signals, which were recorded using a camera.

### 4.7. Yeast Two-Hybrid Verification

Proteins that interact with DELLA proteins were predicted using STRING (STRING: functional protein association networks (string-db.org)). The *PfGRAS12/PfJAZ3* genes were amplified using specific primers, and the yeast two-hybrid vectors PGBKT7-*PfGRAS12* and PGADT7-*PfJAZ3* were constructed and transformed into yeast strain YH109. Double digestion of PGADT7 and PGBKT7 vectors used FD-ECOR1/Bamhl and FD-Ndel/ ECOR1, respectively. After detecting self-activation [25], each of the strains was plated separately on SD/-Leu-Trp and SD/-Trp-Leu-His-Ade + 70 mM 3-aminotriazole selection media and cultured at 30 °C for 3–4 days.

### 4.8. Bimolecular Fluorescence Complementation

Primers for the pNC-*PfGRAS12*-ECN and pNC-*PfJAZ3*-ENN vectors were designed and synthesized using a Nimble Cloning kit according to the manufacturer’s instructions. The ECN/ENN vector contains an eYFP tag for fluorescence microscopy. The PGBKT7-*PfGRAS12* and PGADT7-*PfJAZ3* plasmids were used as templates and were ligated with pNC-BiFC-ECN and pNC-BiFC-ENN vectors, which were then transformed into GV3101-psoup-P19. The resulting constructs were examined using laser confocal laser scanning microscopy to verify the interactions between the PfGRAS12 and PfJAZ3 proteins in *P. fortunei*.

## 5. Conclusions

In conclusion, *P. fortunei* plays an important role in China’s agroforestry and eco-pharmacology; is widely used in paper, furniture, musical instruments, construction and other fields [38]; and is also an important antibacterial, anti-inflammatory, and antioxidant agent [39]. However, the occurrence of phytophthora infestation has seriously affected the development of China and even the world’s *P. fortunei* industry. Under the objective condition that it is difficult to culture phytoplasma in vitro and the opportunity of the rapid development of sequencing technology, it is the focus of the current research on defending against botrytis cinerea and cultivating resistant varieties from the direction of molecular biology to clarify the relationship between PaWB phytoplasma infestation and plant response, and hormone imbalance and protein interaction pathways. In the study of the relationship between gibberellins and dwarfing symptoms of PaWB, DELLA protein, as the core of plant height control, is of great significance to clarify its protein regulatory pathway for the study of PaWB. The results of this study enriched the genetic information of the GARS gene family in *P. fortunei*, which lays the foundation for the study of the regulation of GARS genes in PaWB, especially the DELLA protein, in the dwarfing symptoms of PaWB. It provides a theoretical basis for guiding PaWB breeding and forest improvement.

## Data Availability

Data are contained within the article and Supplementary Materials.

## Supplementary Materials

The following supporting information can be downloaded at: https://www.mdpi.com/article/10.3390/ijms25042425/s1.
