# Supplementary material for: Comprehensive Analysis of the GRAS Gene Family in Paulownia fortunei and the Response of DELLA Proteins to Paulownia Witches’ Broom
Source: Int J Mol Sci. 2024 Feb 19;25(4):2425. doi: 10.3390/ijms25042425 (PMC10888722; doi:10.3390/ijms25042425)
Supplement: Supplementary file 1 [file ijms-25-02425-s001.zip › ijms-2847686-supplementary.pdf]

International Journal of
Molecular Sciences

MDPI

Table S1. Basic characteristics of proteins encoded by *pfGRASs*.

Gene	Accession number	Peptide length(aa）	pI	MW(D)	Atomic composition	Instability Index	Aliphatic index	Subcellular location
PfGRAS1	Pfo02g003840.1	801	5.85	86249.06	$C_{3820}H_{5952}N_{1060}O_{1174}S_{23}$	59.38	80.24	Nucleus
PfGRAS2	Pfo03g010120.1	454	5.74	50216.4	$C_{2230}H_{3501}N_{611}O_{663}S_{23}$	43.87	93.18	Chloroplast
PfGRAS3	Pfo03g013780.1	761	7.65	86379.74	$C_{3834}H_{5974}N_{1074}O_{1150}S_{27}$	41.42	75.45	Nucleus
PfGRAS4	Pfo04g009860.1	474	5.91	53072.15	$C_{2365}H_{3749}N_{639}O_{696}S_{25}$	59.8	95.9	Nucleus
PfGRAS5	Pfo04g012510.1	789	5.65	85326.27	$C_{3787}H_{5878}N_{1038}O_{1155}S_{28}$	55.6	80.7	Nucleus
PfGRAS6	Pfo05g004830.1	477	6.05	53127.13	$C_{2354}H_{3653}N_{665}O_{701}S_{20}$	45.78	83.21	Endoplasmic reticulum
PfGRAS7	Pfo05g007100.1	471	5.95	53050.12	$C_{2363}H_{3739}N_{641}O_{693}S_{26}$	58.43	91.96	Cytoplasm
PfGRAS8	Pfo05g008820.1	336	5.36	37553.59	$C_{1685}H_{2674}N_{444}O_{487}S_{19}$	49.21	108.3	Chloroplast
PfGRAS9	Pfo05g010890.1	591	8.91	67380.34	$C_{2986}H_{4676}N_{842}O_{896}S_{21}$	44.25	70.92	Nucleus
PfGRAS10	Pfo05g010900.1	719	6.55	81560.98	$C_{3569}H_{5555}N_{1025}O_{1091}S_{40}$	56.63	66.84	Nucleus
PfGRAS11	Pfo05g010910.1	756	5.07	85015.49	$C_{3770}H_{5844}N_{1018}O_{1174}S_{25}$	52.08	77.22	Nucleus
PfGRAS12	Pfo06g003490.1	582	4.92	63212.32	$C_{2768}H_{4355}N_{767}O_{871}S_{28}$	34.94	82.98	Nucleus
PfGRAS13	Pfo06g007940.1	606	5.14	66552.01	$C_{2968}H_{4607}N_{793}O_{913}S_{17}$	54.69	80.23	Nucleus
PfGRAS14	Pfo06g008150.1	554	5.79	61474.81	$C_{2758}H_{4303}N_{749}O_{819}S_{13}$	44.7	90.63	Chloroplast
PfGRAS15	Pfo06g010090.1	505	5.63	57037.9	$C_{2505}H_{3870}N_{710}O_{775}S_{22}$	50.92	72.02	Nucleus
PfGRAS16	Pfo06g010290.1	452	5.68	50464.72	$C_{2251}H_{3517}N_{613}O_{662}S_{22}$	42.98	89.98	Chloroplast
PfGRAS17	Pfo06g012740.1	420	6.57	46705.47	$C_{2066}H_{3272}N_{586}O_{609}S_{20}$	36.52	89.64	Chloroplast
PfGRAS18	Pfo07g006000.1	586	5.12	64178.3	$C_{2833}H_{4402}N_{778}O_{876}S_{25}$	48.95	79.71	Nucleus
PfGRAS19	Pfo07g009710.1	736	5.57	80472.42	$C_{3593}H_{5567}N_{967}O_{1073}S_{31}$	44.68	87.18	Cytoplasm
PfGRAS20	Pfo07g011240.1	674	6.13	75003.88	$C_{3294}H_{5201}N_{945}O_{1006}S_{27}$	61.6	80.42	Nucleus
PfGRAS21	Pfo08g007560.1	424	5.54	46786.48	$C_{2081}H_{3283}N_{569}O_{621}S_{18}$	50.25	97.73	Cytoplasm
PfGRAS22	Pfo08g014150.1	554	6.38	61385.63	$C_{2691}H_{4195}N_{777}O_{838}S_{17}$	43.88	72.37	Nucleus
PfGRAS23	Pfo09g008150.1	553	5.51	61286.71	$C_{2689}H_{4178}N_{750}O_{844}S_{24}$	50.89	76.5	Nucleus
PfGRAS24	Pfo09g009840.1	426	6.44	47956.5	$C_{2148}H_{3350}N_{612}O_{620}S_{9}$	57.33	96	Cytoplasm
PfGRAS25	Pfo09g012760.1	584	6.04	65429.43	$C_{2876}H_{4476}N_{824}O_{885}S_{21}$	56.98	76.5	Nucleus
PfGRAS26	Pfo09g019200.1	343	6.45	37467.84	$C_{1656}H_{2647}N_{467}O_{497}S_{13}$	46.2	99.04	Chloroplast
PfGRAS27	Pfo09g020030.1	547	5.51	61065.1	$C_{2687}H_{4184}N_{740}O_{826}S_{31}$	49.92	81.3	Chloroplast
PfGRAS28	Pfo10g000470.1	728	5.59	80700.04	$C_{3548}H_{5559}N_{995}O_{1097}S_{31}$	54.44	80.91	Nucleus
PfGRAS29	Pfo10g003570.1	568	6.25	61865.8	$C_{2700}H_{4308}N_{768}O_{854}S_{21}$	57.74	79.47	Chloroplast
PfGRAS30	Pfo10g009350.1	746	5.94	84404.66	$C_{3733}H_{5823}N_{1027}O_{1135}S_{36}$	47.23	74.46	Nucleus
PfGRAS31	Pfo10g009370.1	746	5.94	84404.66	$C_{3733}H_{5823}N_{1027}O_{1135}S_{36}$	47.23	74.46	Nucleus
PfGRAS32	Pfo11g000660.1	324	5.12	35874.89	$C_{1578}H_{2514}N_{416}O_{499}S_{18}$	52.68	82.41	Nucleus
PfGRAS33	Pfo11g000670.1	658	8.22	74888.78	$C_{3340}H_{5286}N_{914}O_{956}S_{42}$	47.24	91.13	Cytoplasm
PfGRAS34	Pfo11g000680.1	447	7.85	50853.79	$C_{2254}H_{3601}N_{615}O_{665}S_{28}$	36.11	92.04	Nucleus
PfGRAS35	Pfo11g000690.1	487	6.01	55157.38	$C_{2472}H_{3878}N_{656}O_{725}S_{24}$	38.43	90.51	Nucleus
PfGRAS36	Pfo11g006510.1	453	5.34	50925.71	$C_{2229}H_{3520}N_{620}O_{695}S_{25}$	50.36	79.38	Nucleus
PfGRAS37	Pfo11g013890.1	567	5.05	63406.97	$C_{2763}H_{4320}N_{774}O_{885}S_{27}$	52.04	78.59	Nucleus
PfGRAS38	Pfo11g015060.1	462	6.16	52755.26	$C_{2359}H_{3677}N_{629}O_{700}S_{22}$	42.01	82.28	Nucleus

PfGRAS39	Pfo12g000210.1	579	5.3	65041.27	$C_{2883}H_{4525}N_{781}O_{868}S_{32}$	43.33	86.07	Cytoplasm
PfGRAS40	Pfo12g004460.1	470	5.65	52950.02	$C_{2354}H_{3731}N_{633}O_{697}S_{28}$	53.26	93.2	Endoplasmic reticulum
PfGRAS41	Pfo13g002280.1	407	5.57	44455.69	$C_{1976}H_{3095}N_{545}O_{587}S_{18}$	33.49	90.64	Cytoplasm
PfGRAS42	Pfo13g005740.1	602	5.21	66028.23	$C_{2943}H_{4542}N_{784}O_{909}S_{18}$	49.8	77.62	Nucleus
PfGRAS43	Pfo13g005930.1	552	5.64	61135.22	$C_{2736}H_{4238}N_{746}O_{815}S_{16}$	49.9	85.34	Chloroplast
PfGRAS44	Pfo13g007670.1	505	5.63	56864.79	$C_{2498}H_{3843}N_{701}O_{771}S_{26}$	51.55	68.55	Nucleus
PfGRAS45	Pfo13g012150.1	539	6.64	60368.51	$C_{2671}H_{4192}N_{748}O_{805}S_{22}$	58.25	83.81	Nucleus
PfGRAS46	Pfo14g005090.1	510	6.13	56216.55	$C_{2505}H_{3956}N_{684}O_{737}S_{24}$	52.67	95.99	Cytoplasm
PfGRAS47	Pfo14g007210.1	176	4.81	19777.53	$C_{879}H_{1373}N_{235}O_{266}S_{9}$	39.15	92.51	Nucleus
PfGRAS48	Pfo15g001820.1	461	7.58	52682.36	$C_{2349}H_{3690}N_{636}O_{694}S_{23}$	38.52	81.59	Nucleus
PfGRAS49	Pfo15g002590.1	487	5.62	54178.94	$C_{2378}H_{3690}N_{678}O_{729}S_{23}$	38.2	80.33	Nucleus
PfGRAS50	Pfo15g003740.1	576	5.16	64517.12	$C_{2816}H_{4399}N_{785}O_{904}S_{25}$	49.37	77.53	Nucleus
PfGRAS51	Pfo16g000130.1	411	5.58	46240.19	$C_{2072}H_{3237}N_{557}O_{599}S_{22}$	43.1	94.02	Cytoskeleton
PfGRAS52	Pfo16g000660.1	537	6.39	59135.51	$C_{2648}H_{4152}N_{726}O_{777}S_{17}$	61.94	90.5	Chloroplast
PfGRAS53	Pfo16g006870.1	583	5.59	65180.26	$C_{2876}H_{4457}N_{803}O_{885}S_{23}$	54.24	78.81	Chloroplast
PfGRAS54	Pfo16g008010.1	539	4.64	59853.69	$C_{2634}H_{4122}N_{716}O_{821}S_{28}$	45.29	90.99	Cytoplasm
PfGRAS55	Pfo16g008480.1	427	6.31	47895.51	$C_{2146}H_{3351}N_{607}O_{620}S_{10}$	62.78	96.95	Cytoplasm
PfGRAS56	Pfo16g009370.1	538	5.39	59352.32	$C_{2601}H_{4103}N_{719}O_{809}S_{30}$	51.62	89.74	Nucleus
PfGRAS57	Pfo16g010020.1	552	5.94	61825.67	$C_{2701}H_{4212}N_{770}O_{835}S_{31}$	54.65	78.04	Nucleus
PfGRAS58	Pfo17g000310.1	563	5.24	61714.8	$C_{2721}H_{4259}N_{745}O_{842}S_{26}$	49.33	82.9	Nucleus
PfGRAS59	Pfo19g001000.1	587	5.11	65750.68	$C_{2907}H_{4524}N_{770}O_{896}S_{36}$	44.29	78.4	Nucleus
PfGRAS60	Pfo19g001010.1	565	5.11	63770.46	$C_{2844}H_{4466}N_{752}O_{871}S_{20}$	48.8	89.2	Nucleus
PfGRAS61	Pfo19g001020.1	556	5.65	63332.24	$C_{2814}H_{4440}N_{762}O_{851}S_{24}$	52.99	87.51	Nucleus
PfGRAS62	Pfo19g001030.1	579	6.31	64383.77	$C_{2853}H_{4558}N_{778}O_{864}S_{25}$	44.07	95.19	Cytoplasm
PfGRAS63	Pfo19g001040.1	451	5.53	50852.25	$C_{2236}H_{3554}N_{612}O_{682}S_{29}$	42.18	89.71	Nucleus
PfGRAS64	Pfo19g008150.1	403	5.42	45037.2	$C_{1984}H_{3170}N_{548}O_{616}S_{15}$	41.6	92.86	Cytoplasm
PfGRAS65	Pfo20g004210.1	747	6.39	84028.06	$C_{3715}H_{5799}N_{1039}O_{1126}S_{31}$	44.54	75.29	Nucleus
PfGRAS66	Pfo20g008370.1	625	6.78	67856.72	$C_{2950}H_{4728}N_{846}O_{934}S_{27}$	55.48	79.04	Chloroplast
PfGRAS67	Pfoxxg000700.1	680	5.83	73398.44	$C_{3226}H_{5084}N_{904}O_{1016}S_{19}$	52.62	82.68	Chloroplast
PfGRAS68	Pfoxxg009460.1	209	5.55	23455.2	$C_{1046}H_{1595}N_{287}O_{319}S_{5}$	65.25	66.25	Nucleus
PfGRAS69	Pfoxxg009470.1	765	6.32	87157.81	$C_{3865}H_{6013}N_{1075}O_{1158}S_{34}$	45.9	76.61	Nucleus
PfGRAS70	Pfoxxg010850.1	386	5.08	44663.19	$C_{2000}H_{2983}N_{529}O_{595}S_{22}$	43.95	69.38	Nucleus
PfGRAS71	Pfoxxg010860.1	522	4.73	58963.64	$C_{2585}H_{4031}N_{693}O_{816}S_{34}$	55.8	77.5	Nucleus
PfGRAS72	Pfoxxg011760.1	425	5.78	46540.32	$C_{2050}H_{3240}N_{580}O_{640}S_{9}$	52.05	86.6	Nucleus
PfGRAS73	Pfoxxg012780.1	792	6.06	85114.36	$C_{3721}H_{5861}N_{1059}O_{1180}S_{25}$	50.81	78.62	Nucleus
PfGRAS74	Pfoxxg014180.1	755	6.41	86196.66	$C_{3828}H_{5957}N_{1065}O_{1146}S_{30}$	45.36	77.49	Nucleus
PfGRAS75	Pfoxxg014190.1	465	9.18	52896.73	$C_{2380}H_{3724}N_{660}O_{675}S_{16}$	45.95	82.22	Cytoplasm
PfGRAS76	Pfoxxg020540.1	512	4.74	57715.28	$C_{2527}H_{3950}N_{680}O_{798}S_{34}$	56.33	78.45	Nucleus
PfGRAS77	Pfoxxg021460.1	527	8.07	56156.67	$C_{2417}H_{3854}N_{710}O_{791}S_{20}$	52.93	71.33	Nucleus
PfGRAS78	Pfoxxg024740.1	792	6.06	85114.36	$C_{3721}H_{5861}N_{1059}O_{1180}S_{25}$	50.81	78.62	Nucleus
PfGRAS79	Pfoxxg025380.1	530	4.87	61051.65	$C_{2733}H_{4093}N_{713}O_{822}S_{30}$	47.66	71.47	Peroxisome

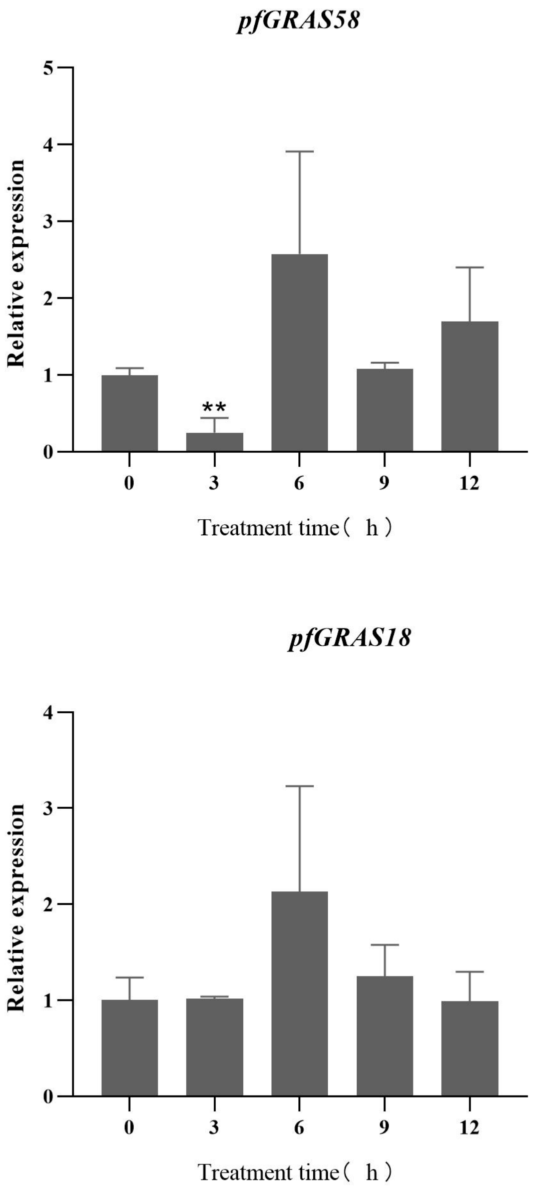

Figure S1. Expression patterns of *PfGRAS18/58* under GA3 treatment.

Table S2. Primer sequence information.

Gene	Forward primer (5'-3')	Reverse primer (5'-3')
PfGRAS9	TGGAGCAACCACACTTCACA	GAATCCCGGTTGGGGGAAAT
PfGRAS12	TTCGAGTTCCGTGGCTTTGT	CAGCACTTTGTCGATGGCAC
PfGRAS15	GGACTATGTTCGGGCACGTT	CAAATGCGGGGTTTCATCGG
PfGRAS18	AGTGAGGTTTACTTGGGGCG	TATACCCATCCCCACCAGCA
PfGRAS19	AGCCTCCTGCGGATCTTTTT	TGCTGCTGGATACTCACACC
PfGRAS23	AGACAGGTTGCAGTATCGGC	GGATAACAACCTCGCCCTGA
PfGRAS41	AGCTTCACCGGCTGTTATCC	TTCGCCTCCTGCTCAACAAT
PfGRAS58	TGGTTGACTCGCAGGAGAAC	TCTCATAGCTCCTGCTTGCG
PfActin	AATGGAATCTGCTGGAAT	ACTGAGGACAATGTTACC
PfGRAS12- PSAK277	GTGGATCCAAAGAATTCATGAAAAGAGATCGTGATCG	CTCCTTTACCCATGAATTCTTCCCCTGCCGTGAGCCGCC
PfGRAS12-ECN	AGTGGTCTCTGTCCAGTCCTATGAAAAGAGATCGTGATCG	GGTCTCAGCAGACCACAAGTTTCCCCTGCCGTGAGCCGCC
PfJAZ3-ENN	AGTGGTCTCTGTCCAGTCCTTGGAGAGAGATTTCATGGG	GGTCTCAGCAGACCACAAGTATTGATTGCTGGAACAGGAC
PfGRAS12-BD	GAGGAGGACCTGCATATGATGAAAAGAGATCGTGATCG	ACGGATCCCCGGGAATTCTTCCCCTGCCGTGAGCCGCC
PfJAZ3-AD	GCCATGGAGGCCAGTGAATTCATGGAGAGAGATTTCATGGG	CAGCTCGAGCTCGATGGATCCATTGATTGCTGGAACAGGAC
